# Bone Marrow Granuloma in Typhoid Fever: A Morphological Approach and Literature Review

**DOI:** 10.1155/2015/628028

**Published:** 2015-02-19

**Authors:** Kavitha Muniraj, Somanath Padhi, Manjiri Phansalkar, Periyasami Sivakumar, Renu G'Boy Varghese, Reba Kanungo

**Affiliations:** ^1^Department of Pathology, Pondicherry Institute of Medical Sciences, Puducherry 605014, India; ^2^Department of General Medicine, Pondicherry Institute of Medical Sciences, Puducherry 605014, India; ^3^Department of Microbiology, Pondicherry Institute of Medical Sciences, Puducherry 605014, India

## Abstract

Typhoid fever is one of the few bacterial infections in humans where bone marrow evaluation is routinely recommended. However, the morphological aspect of typhoid fever in bone marrow has been rarely described in the literature. We describe a 25-year-old male patient who presented with prolonged fever suspected to be of tubercular etiology. Bone marrow examination showed well-formed histiocytic and epithelioid granulomas and erythrophagocytosis; and the bone marrow aspirate culture grew *Salmonella typhi* A. In view of potential clinical implications, typhoid fever should be considered as a differential diagnosis to tuberculosis in the evaluation of prolonged fever; especially in high prevalent areas. We suggest that erythrophagocytosis may serve as a morphological marker in typhoid granulomas in the bone marrow; and bone marrow culture should be submitted in every suspected case for appropriate patient management.

## 1. Introduction

Bone marrow granuloma (BMG) is not a frequent finding in a bone marrow biopsy. The incidence ranges from 0.3% to 2.2%, depending on the series. The etiologies of BMG are numerous, ranging from infections to inflammatory disorders to hematolymphoid neoplasms, commonest being tuberculosis in India [1, 2]. In spite of high prevalence in developing countries, typhoid fever is among the rare reported causes of BMG. However, the morphological characterization of typhoid fever in bone marrow has been rarely described in the literature [3–5]. Furthermore, differentiating typhoid BMG from that of tuberculosis, in the absence of culture correlation, may lead to initiation of long duration, toxic antitubercular therapy (ATT) [6–9]. In this paper, we describe a case of BMG in a culture proven case of typhoid fever with special emphasis on the morphological differentials with a brief review on the relevant literature. Furthermore, the significance of bone marrow culture correlation in all patients with prolonged fever is also briefly highlighted.

## 2. Case Presentation

A 25-year-old Indian male, student by occupation, presented with 30-day history of fever and 15-day history of dry cough. Patient was apparently normal 30 days back when he developed fever, initially of low grade and then progressed to high grade, associated with chills and rigor which was relieved with medications intermittently. He gave a history of irregular usage of antibiotics and antipyretics at an outside hospital before being finally referred to our hospital for evaluation of pyrexia. He also had episodes of vague abdominal discomfort few days back and his bladder and bowel habits were normal. He belonged to a region in South India known to be endemic for scrub typhus and dengue and gave no history of recent travel, sexual contact, or any contact with patients with tuberculosis.

Physical examination revealed an average built, febrile patient (oral temperature of 102°F), with pulse rate of 100/min with regular rhythm, blood pressure of 110/80 mmHg, and mild conjunctival pallor, but no scleral icterus, superficial lymphadenopathy, hepatosplenomegaly, rash, eschar, or any bony tenderness. Systemic examination revealed no significant abnormalities except for mild tenderness over right hypochondrium. His radiological evaluation was unremarkable except for mild hepatosplenomegaly.

Routine laboratory work-up revealed normocytic normochromic anemia (hemoglobin (Hb): 110 g/L (ref. 140–160 g/L)), mild leukocytosis (leukocyte count (TLC): 11.5 × 10^9^/L (ref. 4–9 × 10^9^/L)), with neutrophilia, raised erythrocyte sedimentation rate (ESR) (72 mm/1st hr (ref. up to 30 mm/1st hr, Westergren)), and raised C-reactive protein of 36 mg/dL (ref. < 6 mg/dL). His liver transaminases and renal function tests were within normal limits; and stool for occult blood test was negative. Microbiological work-up for pyrexia was negative for HIV, HBV surface antigen, HCV, dengue,* Leptospira*, brucellosis, scrub typhus, and malaria parasite. His urine and CSF cultures were sterile; and skin purified protein derivative (PPD) test was nonreactive. Rising Widal titers of* Salmonella typhi* O and H (>1 : 320) were suggestive of* Salmonella typhi* infection.

**Figure 1:**
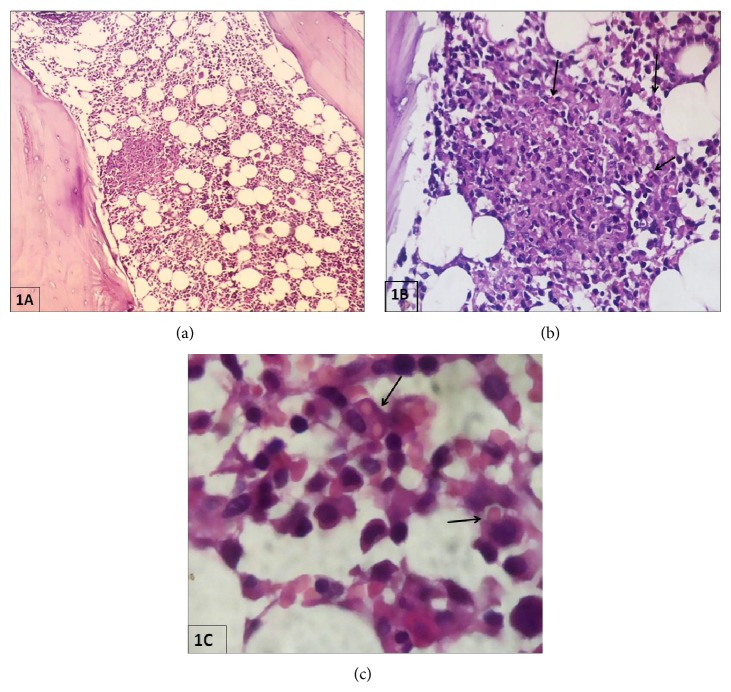
Paraffin embedded decalcified bone marrow trephine biopsy sections from the present case with culture proven typhoid fever which showed epithelioid and histiocytic granulomas in the interstitial and paratrabecular loci. Note the presence of erythrophagocytosis by benign histiocytes (black arrow) in the granulomas (Hematoxylin eosin stain: 100x (a), 400x (b), and 1000x (c)).

Bone marrow aspirate smears from right posterior superior iliac spine were hypercellular (for age) and showed normoblastic erythroid maturation, granulocytic hyperplasia (myeloid to erythroid ratio = 6 : 1), increased megakaryocytes, plasma cells (8%), and relative prominence of histiocytes (5%) with engulfed debris and evidence of erythrophagocytosis. There was no evidence of granuloma, hemoparasites, or malignancy; and stains for* Mycobacterium tuberculosis* and fungi were negative. Paraffin embedded decalcified sections of the bone marrow trephine biopsy (hematoxylin-eosin, periodic acid Schiff) revealed an average cellularity of 70% and maintained hematopoiesis with mild dyspoietic change and multiple well-formed noncaseating granulomas in interstitial and paratrabecular loci. These granulomas showed aggregates of benign histiocytes with foamy cytoplasm and reniform vesicular nuclei (some showing erythrophagocytosis), epithelioid cells, admixed with scattered lymphocytes and plasma cells. There was no evidence of Langhans giant cells, acid fast bacilli, fungi, or any malignancy (Figures 1(a), 1(b), and 1(c)). Thus, considering the presence of BMGs exhibiting erythrophagocytosis, possibility of typhoid granuloma was suspected. Bone marrow aspirate was sent to the clinical microbiology department for culture. The material was inoculated into BacT-Alert bottle and incubated in the automated culture system (Bio Meiurex). Following a beep within 8 hours, a subculture onto 5% sheep blood agar showed moist colonies. This was identified by standard biochemical tests and serotyped using specific antisera that is polyvalent O and factor 9D. It was confirmed as* Salmonella enterica* var Typhi A (*Salmonella typhi* A). Antibiotic susceptibility was done on Mueller-Hinton agar for ceftriaxone, cotrimoxazole, chloramphenicol, and ciprofloxacin. Results were interpreted as per the Clinical Laboratory Standards Institute (CLSI-2013) guidelines [10]. The patient responded well to treatment with ceftriaxone and metronidazole and was finally discharged with satisfactory outcome. The informed consent was obtained from the next of kin of the patient.

## 3. Discussion

*Salmonella typhi* is a Gram negative bacillus which, after its colonization in the terminal ileum, bypasses the neutrophil mediated phagocytosis, and enters the Peyer's patches, where it coopts for the host macrophages' cellular machinery for its own reproduction as it is carried through the mesenteric lymph nodes to the thoracic duct and then to the reticuloendothelial tissues in the liver, spleen, and bone marrow. Cell culture and murine model studies have recently shown that* Salmonella* species reside within the hemophagocytic macrophages in persistent infection. These cells (so-called “typhoid cells”) may provide a survival advantage to these organisms and serve as clinical marker of typhoid fever [11].

The protean manifestations of typhoid fever make this disease a true diagnostic challenge. The classic presentations of stepladder pattern pyrexia, malaise, diffuse abdominal pain, and constipation (resulting from Peyer patch hyperplasia and narrowing of the intestinal lumen) are increasingly uncommon nowadays and more often are reported from endemic countries such as India. Young children, individuals with AIDS, and one-third of immunocompetent adults who develop typhoid fever develop diarrhea rather than constipation. Untreated, typhoid fever is a grueling illness that may progress to delirium, obtundation, intestinal hemorrhage, bowel perforation, and death within 1 month of onset. Survivors may be left with long-term or permanent neuropsychiatric complications [12]. Our patient had vague diffuse abdominal pain with irregular episodes of fever (since last 1 month), mild hepatosplenomegaly, neutrophilic leukocytosis (rather than neutropenia) and tachycardia (rather than bradycardia) at presentation, and cough which led to a suspicion of tuberculosis; and therefore bone marrow evaluation was done for evaluation of pyrexia before initiation of empiric ATT.

There has been paucity of the literature regarding the association and morphological characterization of BMGs in typhoid fever (Table 1) [3–9]. Shin et al. [3] reported the bone marrow morphology in 16 patients (age group: 20–56 years, M : F = 7 : 8) with culture proven typhoid fever and stratified them as per the disease stage. The most frequent finding was chronic granulomatous inflammation (*n* = 8, well formed in 4, ill-defined in 4). Four (25%) had infection (bacteria) associated hemophagocytic lymphohistiocytosis (HLH), whereas reactive changes and myeloid hyperplasia were described in two patients each. Granulocytic hyperplasia with hemophagocytosis appeared in the early stage of the infection; histiocytic hyperplasia, ill-formed granulomas, and erythrophagocytosis were more apparent during the intermediate/proliferative stage, whereas well-formed histiocytic/epithelioid granulomas with prominent erythrophagocytosis were characteristically seen more in the late/lytic phase of the disease. Another large Korean study [4] described the bone marrow findings in 60 patients with typhoid fever (age group: 10–60 years; 34/60 culture proven, 10/60 serologically proven, and 16/60 clinically suspected). Eight of 49 (16%) cases had BMGs on trephine biopsy, of which six were of histiocytic nature and two were of epithelioid type. In addition, the “typhoid cells” (histiocytes with engulfed debris/erythrocytes) were identified in the bone marrow aspirate smears in nearly half of all cases. Though there was no specific morphology of BMGs, there was a high yield of organisms in the bone marrow aspirate culture. Similarly, as described by Lee et al. [5] in their 27 cases, presence of histiocytic and/or epithelioid BMGs with engulfed debris and/or erythrophagocytosis, lack of caseous necrosis, and Langhans giant cells were more in favor of typhoid fever than tuberculosis. Sakhalkar et al. [6] reported a 2-year-old Down's syndrome child with typhoid granuloma in bone marrow (culture proven) who presented with fever, hepatomegaly, and pancytopenia; and BMGs with engulfed debris and erythrophagocytosis/hemophagocytosis were described to be the morphological hallmark of the disease. Mert et al. [7] reported the first case (in the English literature) of combined splenic, hepatic, and bone marrow granulomas in a patient with typhoid fever. Lee et al. [8] reported the first case of focal necrotizing epithelioid BMG in a Korean male with culture proven* Salmonella paratyphi* A, though hemophagocytosis was not detected. Bharadwaj et al. [9] described another case of typhoid granulomas (both necrotizing and nonnecrotizing) in the mesenteric lymph node with giant cells and occasional erythrophagocytosis. While the three large Korean studies [3–5] did not describe the outcome in their patients, all the above 4 reported patients [6–9] had a favorable outcome following initiation of definitive antibiotic therapy. In accordance with previous studies, the presence of so-called “typhoid cells” in the bone marrow aspirate as well as histiocytic and epithelioid granulomas with erythrophagocytosis was the clue in elucidating the exact etiology. Besides this, culture correlation was instrumental in avoiding the initiation of empiric antitubercular therapy.

**Figure 2:**
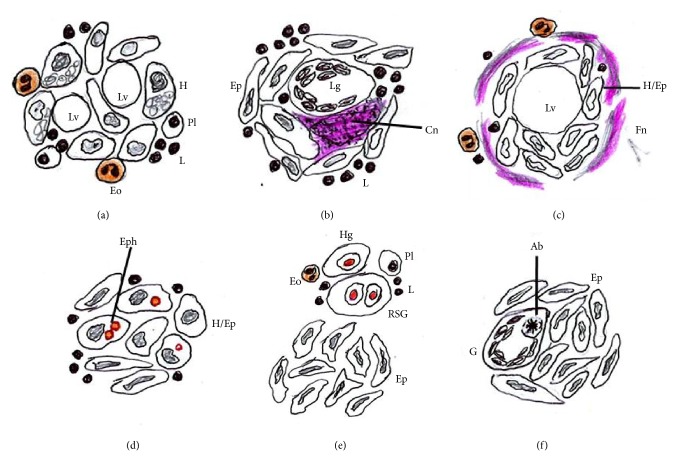
Schematic line representation of various morphological patterns of bone marrow granulomas associated with different etiologies. (a) Lipid granuloma commonly described in the earlier literature, (b) tubercular caseating epithelioid granuloma with Langhans giant cell, (c) fibrin ring/“doughnut” granuloma seen in Q-fever, brucellosis, and sometimes in viral infections, (d) histiocytic/epithelioid granuloma with erythrophagocytosis in typhoid fever, (e) epithelioid granuloma seen in Hodgkin lymphoma and surrounding atypical mononuclear cells and polymorphous inflammatory infiltrate, and (f) naked, noncaseating (sarcoidal type) epithelioid granuloma seen in sarcoidosis. Ab: asteroid body, Cn: caseous necrosis with eosinophilic, granular appearance; Eo: eosinophils; Ep: epithelioid histiocytes with characteristic elongated, slipper shaped nuclei; Eph: erythrophagocytosis; Fn: fibrinoid necrosis/material; G: multinucleated giant cell; H: foamy histiocytes with or without engulfed debris; Hg: mononuclear Hodgkin cell with eosinophilic macro nucleoli; L: lymphocytes; Lv: lipid vacuole; Lg: Langhans giant cell; Pl: plasma cells; RSG: Reed-Sternberg giant cell characterized by mirror image nuclei with eosinophilic macro nucleoli.

The presence of BMG in the trephine biopsies is a nonspecific finding and requires correlation with clinical presentation and drug history, as well as a battery of microbiological, serological, autoimmune work-up. Considering various etiologies, the morphological characteristics of BMG may be the only clue to reach a most probable underlying cause. A schematic approach to the morphological diagnosis of BMGs in different conditions is presented in Figure 2. Morphologically, BMGs are broadly classified into caseating or noncaseating (sarcoidal type) epithelioid granuloma, lipid granuloma, histiocytic or epithelioid granuloma with erythrophagocytosis, doughnut or fibrin ring granuloma, and so forth. Lipid granulomas are relatively common nonspecific findings in bone marrow sections and are characterized by their close proximity to sinusoids or lymphoid nodules, smaller size (0.2 to 0.8 micron), central lipid vacuole admixed with variable number of lymphocytes, plasma cells, and eosinophil. “Doughnut” or fibrin ring granuloma is a subtype of epithelioid granuloma characterized by a central fat vacuole and annular peripheral fibrinoid material, seen more commonly in association with Q fever, viral infections (EBV, CMV, and hepatitis A), and rarely with patients with Hodgkin/peripheral T cell lymphomas (HL/PTCL) and allopurinol induced hepatitis [13]. Well-formed or ill-formed caseating epithelioid granulomas, Langhans giant cells, and inflammatory cells are the usual features of bone marrow involvement in disseminated tuberculosis. Well-formed, naked, noncaseating, reticulin-rich, epithelioid granulomas with giant cells containing cytoplasmic asteroid and/or Schaumann bodies are more in favor of marrow involvement in sarcoidosis. Similarly, variably sized histiocytic and/or epithelioid granulomas with or without reticulin fibrosis may be the initial manifestation of marrow involvement in HL/PTCL; and presence of atypical mononuclear Hodgkin-like cells in a polymorphous background may aid in the diagnosis. In contrast and as described in the literature [3–9], erythrophagocytosis/hemophagocytosis in the granulomas may be the only clue to the diagnosis of typhoid fever as was evident in the present case.

## 4. Conclusion

Typhoid fever is one of the bacterial infections of humans for which bone marrow examination is routinely recommended, and the yield of bone marrow aspirate culture has been found to be superior to blood, rectal swab, or stool culture in the diagnosis of typhoid fever [14]. Typhoid fever should be considered as a first differential diagnosis to tuberculosis in the evaluation of bone marrow granulomas in patients presenting with prolonged fever, especially in more prevalent areas, as well as those with a history of travel to endemic countries. Erythrophagocytosis in the granulomas may be a helpful feature for correct diagnosis and appropriate management.

## Figures and Tables

**Table 1:** Typhoid granuloma: a brief review of the literature.

Author, year, Reference	Number of cases (*n*)	Age (years)/gender	Site	Pathology	Microbiology	Remarks
Shin et al. [3], 2004	16	20–56, male : female = 7 : 8	Bone marrow	8/16: chronic granulomatous inflammation (4: well-formed, 4: ill-formed) 4/16: HLH 4/16: reactive marrow 2/16: myeloid hyperplasia 2/16: nonspecific Erythrophagocytosis + Phagocytosis of the debris +	*Salmonella typhi* Culture proven (100%)	Early phase: myeloid hyperplasia Proliferative phase-histiocytic hyperplasia, granulomas, and erythrophagocytosis + Lysis phase: well-formed granulomas with erythrophagocytosis +.

Young et al. [4], 1986	60	10–60	Bone marrow	Typhoid cells: 50.8% in BMA; 8/49: granulomas on BMBx (4/8: typhoid cells +) Type of BMG: 6/8: mature histiocytic granuloma, 2/8: epithelioid granuloma Granuloma number: One in 4, two in 3, and four in one	*Salmonella typhi* 34/60: bacteriologically proven; 10/60: serologically proven; 16/60: clinically suspected	No specific morphology of BMG, BMA culture: high yield

Lee et al. [5], 1985	27	—	Bone marrow	Well-formed granuloma (57%), ill formed granuloma (43%) Phagocytosis of nuclear debris, RBCs; ++. No caseation or Langhans giant cells	*Salmonella typhi* Culture proven (100%)	Histiocytic phagocytosis, absence of necrosis and Langhans giant cells, clue to diagnosis of typhoid granulomas

Sakhalkar et al. [6], 2001	1	2, child with Down syndrome	Bone marrow	Fever, pancytopenia, hepatomegaly, hemophagocytosis, BMG	*Salmonella typhi* Culture (BMA and blood)	EBV positive, * Salmonella typhi* +

Mert et al. [7], 2004	2	55, females	Liver and spleen	BMA, NAD Noncaseating granulomas	*Salmonella typhi* (blood)	1st case of typhoid splenic granuloma in the English literature.
		66, males	Bone marrow and liver	2 granulomas in BMBx (noncaseating) 1 microgranuloma in portal region	*Salmonella typhi* Serology Culture (blood, BMA)	

Lee et al. [8], 2004	1	34, males	Bone marrow	Focal epithelioid granuloma Necrotic debris, phagocytosis not seen	*Salmonella paratyphi A* Culture (blood and BMA) Serology (typhi H); + (1 : 320)	—

Bharadwaj et al. [9], 2009	1	47, males	Terminal ileum, mesenteric lymph node	Necrotizing and nonnecrotizing epithelioid granulomas, giant cells present Lymphoerythrophagocytosis + (occasional)	*Salmonella typhi* Serology	—

Present case 2013	1	24, male	Bone marrow	Well-formed noncaseating granulomas (histiocytic and epithelioid type) Erythrophagocytosis ++	*Salmonella typhi* Serology Culture (BMA, blood)	Erythrophagocytosis is a clue to the diagnosis of typhoid granulomas

HLH: hemophagocytic lymphohistiocytosis, +: present, BMA: bone marrow aspiration, BMBx: bone marrow trephine biopsy, BMG: bone marrow granuloma, EBV: Epstein-Barr virus; NAD: no abnormality detected.
